# Maternal Circulating sFlt-1:PlGF Ratio and Stillbirth

**DOI:** 10.1001/jamanetworkopen.2026.7652

**Published:** 2026-04-17

**Authors:** Mi-Sun S. Lee, Ki-Do Eum, David. C. Christiani

**Affiliations:** 1 Department of Environmental Health, Harvard T.H. Chan School of Public Health, Boston, Massachusetts; 2 Ariadne Labs, Brigham and Women’s Hospital and Harvard T.H. Chan School of Public Health, Boston, Massachusetts; 3 Harvard Medical School and Massachusetts General Hospital, Boston

## Abstract

**Importance:**

Stillbirth remains a major global health challenge, particularly in low- and middle-income countries. Angiogenic imbalance, characterized by elevated soluble fms-like tyrosine kinase-1 (sFlt-1) and reduced placental growth factor (PlGF), is implicated in placental dysfunction, yet few studies have examined the association between the sFlt-1:PlGF ratio and stillbirth.

**Objective:**

To investigate the association between maternal circulating sFlt-1:PlGF ratio, using clinically relevant cutoffs, and risk of stillbirth.

**Design, Setting, and Participants:**

This case-control study included participants from 2 health clinics operated by the Dhaka Community Hospital Trust in rural Bangladesh. Pregnant women aged 18 years or older with ultrasonography-confirmed singleton pregnancies were enrolled before or at 16 weeks’ gestation between March 1, 2008, and July 30, 2011. Those delivering stillborn infants (cases) were matched 1:2 with participants in the same cohort who delivered full-term live-born infants (controls). Data were analyzed between September 1, 2025, and January 1, 2026.

**Exposure:**

Maternal serum sFlt-1:PlGF ratio.

**Main Outcomes and Measures:**

The main outcome was stillbirth, defined as fetal death at 28 weeks’ gestation or more. The association between sFlt-1:PlGF ratio cutoffs of greater than 38 and greater than 85 and stillbirth were estimated using Firth penalized logistic regression, with models adjusted using a propensity score incorporating maternal factors.

**Results:**

The analytic sample included 125 participants (mean [SD] age, 22.3 [3.8] years), of whom 39 were included in the stillbirth group and 86 in the live birth control group. After adjusting for propensity score, an sFlt-1:PlGF ratio greater than 38 was significantly associated with an increased odds of stillbirth (odds ratio [OR], 13.44; 95% CI, 2.03-88.89). Similar results were observed using the cutoff of 85 (OR, 12.95; 95% CI, 1.57-106.64). Elevated sFlt-1:PlGF ratios showed high positive predictive value for stillbirth at the cutoff of 38 (88.9%; 95% CI, 51.8%-99.7%) and 85 (85.7%; 95% CI, 42.1%-99.6%). Negative predictive values were moderate at 73.3% (95% CI, 64.3%-81.1%) and 72.0% (95% CI, 63.0%-79.9%), respectively, indicating that low ratios had a limited ability to rule out stillbirth.

**Conclusions and Relevance:**

This case-control study found that a high sFlt-1:PlGF ratio using clinically relevant thresholds was associated with stillbirth and corresponded to an estimated 13-fold increased risk. These findings suggest that angiogenic imbalance may contribute to stillbirth and highlight the potential utility of angiogenic markers as indicators of pregnancy complications in low-resource settings.

## Key Points

**Question:**

Is an elevated ratio of soluble fms-like tyrosine kinase-1 (sFlt-1) to reduced placental growth factor (PlGF) using clinically relevant cutoffs associated with an increased risk of stillbirth?

**Findings:**

In this case-control study of 39 stillbirths and 86 live births, a high sFlt-1:PlGF ratio was significantly associated with a 13-fold greater odds of stillbirth at the clinical cutoffs.

**Meaning:**

These findings suggest that placental angiogenic imbalance may contribute to the pathogenesis of stillbirth in low-resource settings.

## Teaser

This case-control study investigates the association between the ratio of maternal circulating soluble fms-like tyrosine kinase-1 (sFlt-1) to placental growth factor and the risk of stillbirth in Bangladesh.

## Introduction

Stillbirth, also called intrauterine fetal demise, is defined as fetal death based on gestational age thresholds: 28 weeks or more by the World Health Organization (WHO) and 20 weeks or more in the US.^1^ The majority of stillbirths (approximately 98%) occur in low- and middle-income countries (LMICs); however, rates have changed little over the past decade, including in high-income settings.^2^ Despite its high burden with an estimated 2.6 million fetal deaths worldwide and 21 000 annually in the US,^3^ stillbirth remains largely overlooked in policy and research and is recognized as a silent epidemic in global health.^4,5^

As of September 16, 2025, the US National Institutes of Health launched a consortium dedicated to addressing the ongoing burden of stillbirth, of which more than 60% of cases remain unexplained.^6^ Although the causes of stillbirth are not fully elucidated, one recognized mechanism involves placental dysfunction.^7^ Angiogenesis is essential for normal placental development. An imbalance between proangiogenic and antiangiogenic factors, as reflected by an elevated ratio of soluble fms-like tyrosine kinase 1 (sFlt-1) to placental growth factor (PlGF), can impair placental vascularization and maternal-fetal exchange.^8^ While an elevated sFlt-1:PlGF ratio has been associated with preeclampsia, early-onset fetal growth restriction (FGR), and small for gestational age (SGA),^9,10,11,12^ evidence regarding its association with stillbirth remains scarce.

Examining whether angiogenic imbalance contributes to stillbirth is therefore a critical research priority, particularly in low-resource settings in which the burden is greatest and advanced diagnostic tools are limited. Bangladesh is among the top 10 countries with the highest burden of stillbirth, with an estimated 20.4 per 1000 births^13^; however, circulating angiogenic biomarkers in this context are underexplored. To address this research question, we conducted a case-control study within a pregnancy cohort in Bangladesh to evaluate the association between maternal circulating sFlt-1:PlGF ratio, using clinically relevant cutoffs, and the risk of stillbirth.

## Methods

### Study Design and Data

This case-control study was nested within a large, well-characterized pregnancy cohort in Bangladesh.^14,15,16,17^ The study protocol was approved by the human research committees at the Harvard T.H. Chan School of Public Health and the Dhaka Community Hospital Trust. All participants provided written informed consent before enrollment. The study adhered to the Strengthening the Reporting of Observational Studies in Epidemiology (STROBE) reporting guideline.

The parent cohort enrolled pregnant women who were aged 18 years or older with an ultrasonography-confirmed singleton pregnancy at 16 weeks’ gestation or less between March 1, 2008, and July 30, 2011. Cohort participants were followed up at approximately 28 weeks’ gestation, at delivery, and within 4 weeks post partum. Case individuals included women with stillbirth deliveries. Control individuals were randomly selected at a 1:2 ratio from women in the same cohort with full-term live births (ie, excluding those with preterm, low birth weight, SGA, or large for gestational age birth outcomes).

### Measures

Maternal serum samples were collected at approximately 28 weeks’ gestation. Serum sFlt-1 and PlGF levels (in picograms per milliliter) were analyzed using Luminex ×MAP technology (Luminex Corporaton) by an independent laboratory (Eve Technologies). We classified participants as high risk if their sFlt-1:PlGF ratio exceeded 38 or 85 based on prior clinical studies that evaluated these thresholds for prediction and diagnosis of preeclampsia and related adverse pregnancy outcomes.^9,11^

### Covariates

Trained Dhaka Community Hospital health care workers administered structured questionnaires to obtain sociodemographic, lifestyle, and environmental data at enrollment (≤16 weeks’ gestation). Based on previous findings associated with birth outcomes in this cohort,^14,15^ we selected the following a priori covariates: maternal age at enrollment, maternal body mass index (BMI) at enrollment, household income (in taka), secondhand smoke exposure during pregnancy (yes, no), recruitment site (Pabna, Sirajdikhan), and biomass fuel use (fuelwood, crop residues). Maternal height and weight were measured at clinic visits, and BMI was calculated as weight in kilograms divided by height in meters squared.

### Statistical Analysis

The data analysis was performed between September 1, 2025, and January 1, 2026. Descriptive statistics were examined for all variables. For each predefined sFlt-1:PlGF ratio cutoff (>38 and >85), we calculated the proportion of participants exceeding the cutoff within each outcome group (number above cutoff divided by total group size). Differences in these proportions between the stillbirth and live birth groups were assessed using the Fisher exact test given the small cell counts. Due to the small number of stillbirth cases and the potential for confounding, we applied a propensity score adjustment approach. First, we estimated each participant’s propensity of having a high sFlt-1:PlGF ratio using logistic regression, including maternal age at enrollment, BMI, income, secondhand smoke exposure, recruitment site, and biomass fuel use, that was based on prior work in this cohort.^14,15^ The estimated probability from this model was used as the individual propensity score. To estimate the association between sFlt-1:PlGF ratio and stillbirth, we fit a Firth penalized logistic regression model, including both the exposure indicator (sFlt-1:PlGF ratio) and the propensity score.^18^ This complementary approach reduces small sample bias and provides more stable, bias-reduced estimates for rare outcomes, such as stillbirth. The positive predictive value (PPV) and the negative predictive value (NPV) for stillbirth were calculated for the sFlt-1:PlGF ratio at each cutoff. A 2-sided *P* < .05 was considered statistically significant. All statistical analyses were performed using SAS, version 9.4 (SAS Institute, Inc); R, version 4.5.1 (R Foundation for Statistical Computing); and RStudio, version 2026.01.0, build 392 (Posit Software, PBC).

## Results

### Characteristics

Of 1613 participants in the parent cohort, 72 experienced stillbirth deliveries, of whom 46 had serum samples available and were matched 1:2 to 92 control participants from the same cohort with full-term live births. Of these participants, 40 in the stillbirth group and 86 in the life birth group had sufficient serum for the multiplex assay. One participant from the stillbirth group had a PlGF value outside the assay range and was excluded. The analytic sample, therefore, included 125 participants (mean [SD] age, 22.3 [3.8] years; mean [SD] BMI at enrollment, 20.5 [2.9]), with 39 in the stillbirth group and 86 in the live birth group. There were no significant differences between groups in terms of maternal age, household income, secondhand smoke exposure, and recruitment site, but there was a difference in type of biomass fuel used between the live birth and stillbirth groups (crop residues, 19 [22.1%] and 18 [46.2%], respectively; fuelwood, 67 [77.9%] and 21 [53.9%], respectively) (*P* = .007) (Table 1).

**Table 1.** Characteristics of the Study Participants

Characteristic	Participants, No. (%)		*P* value^a^
	Stillbirth group (n = 39)	Live birth group (n = 86)	
Age, mean (SD), y	21.6 (3.7)	22.6 (3.8)	.14
BMI at enrollment, mean (SD)	19.8 (2.1)	20.9 (3.2)	.15
Household income, taka^b^			
≤3000	8 (21.1)	14 (16.3)	.92
3000-4000	10 (26.3)	25 (29.0)	
4001-5000	10 (26.3)	22 (25.6)	
≥5001	10 (26.3)	25 (29.1)	
Secondhand smoke exposure			
Yes	16 (41.0)	25 (29.1)	.19
No	23 (59.0)	61 (70.9)	
Recruitment site			
Pabna	8 (20.5)	32 (37.2)	.06
Sirajdikhan	31 (79.5)	54 (62.8)	
Type of biomass fuel			
Crop residues	18 (46.2)	19 (22.1)	.007
Fuelwood	21 (53.9)	67 (77.9)	

Abbreviation: BMI, body mass index (calculated as weight in kilograms divided by height in meters squared).

^a^ *P* values for statistical differences in covariates between the stillbirth and live birth groups were calculated using *t *test or the Mann-Whitney *U* test for continuous variables, and χ^2^ test for categorial variables.

^b^ One missing in the stillbirth group.

### Comparison of sFlt-1:PlGF Ratio Between the Stillbirth and Live Birth Groups

Using an sFlt-1:PlGF ratio cutoff of 38, 8 participants (20.5%) in the stillbirth group had elevated ratios compared with 1 participant (1.2%) in the live birth group (*P* < .001). Similar patterns were observed with the higher threshold of 85: 6 participants (15.4%) in the stillbirth group exceeded the cutoff vs 1 (1.2%) in the live birth group (*P* = .004) (Figure).

**Figure.**
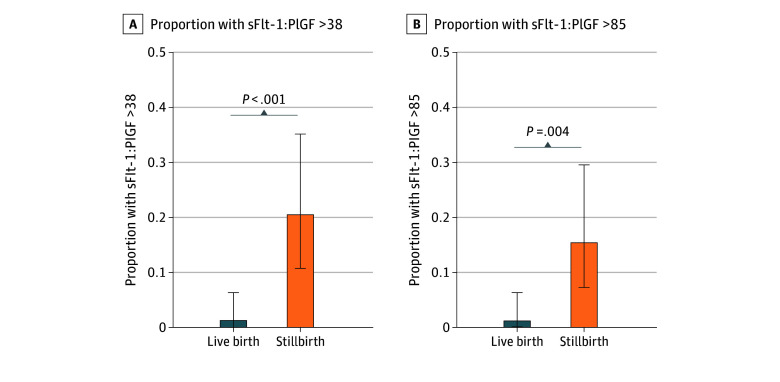
Bar Graph of Ratios of Soluble fms-Like Tyrosine Kinase-1 to Placental Growth Factor (sFlt-1:PlGF) Using Clinical Thresholds Among Stillbirths and Live Births Error bars indicate the Wilson 95% CIs for each proportion.

### Association Between sFlt-1:PlGF Ratio and Stillbirth

Table 2 presents the adjusted association between the sFlt-1:PlGF ratio cutoff and stillbirth risk. Using the sFlt-1:PlGF ratio cutoff of 38, the PPV was 88.9% (95% CI, 51.8%-99.7%), with 8 of 9 participants having an elevated ratio experiencing stillbirth. The NPV was 73.3% (95% CI, 64.3%-81.1%), with 85 of 116 participants with a low ratio (≤38) delivering live births. The higher cutoff of 85 yielded a similar PPV of 85.7% (95% CI, 42.1%-99.6%) and an NPV of 72.0% (95% CI, 63.0%-79.9%). The NPV remained moderate for both cutoffs. An sFlt-1:PlGF ratio greater than 38 was significantly associated with an increased risk of stillbirth (odds ratio [OR], 15.37; 95% CI, 2.36-100.22). This association remained robust after propensity score adjustment based on covariates (OR, 13.44; 95% CI, 2.03-88.89). Similar results were observed when using the higher cutoff of 85, after adjusting for the propensity score (OR, 12.95; 95% CI, 1.57-106.64) (Table 2).

**Table 2.** Adjusted Associations Between sFlt-1:PlGF Ratio Cutoff Values and Risk of Stillbirth

sFlt-1:PlGF ratio cutoff	Participants, No. (%)			OR (95% CI)^a^	PPV, % (95% CI)	NPV, % (95% CI)
	All (N = 125)	Stillbirth group (n = 39)	Live birth group (n = 86)			
>38	9 (7.2)	8 (88.9)	1 (11.1)	13.44 (2.03-88.89)	88.9 (51.8-99.7)	73.3 (64.3-81.1)
≤38	116 (92.8)	31 (26.7)	85 (73.3)	1 [Reference]	NA	NA
>85	7 (5.6)	6 (85.7)	1 (14.3)	12.95 (1.57-106.64)	85.7 (42.1-99.6)	72.0 (63.0-79.9)
≤85	118 (94.4)	33 (28.0)	85 (72.0)	1 [Reference]	NA	NA

Abbreviations: NA, not applicable; NPV, negative predictive value; OR, odds ratio; PPV, positive predictive value; sFlt-1:PlGF, ratio of soluble fms-like tyrosine kinase-1 to placental growth factor.

^a^ Adjusted for a propensity score estimated based on age (in years), body mass index (calculated as weight in kilograms divided by height in meters squared), household income (in taka), secondhand smoke (yes, no), recruitment site (Pabna, Sirajdikhan), and biomass fuel used (crop residues, fuelwood).

## Discussion

This case-control study found that a high sFlt-1:PlGF ratio based on clinically relevant thresholds of greater than 38 and greater than 85 was significantly associated with stillbirth, corresponding to an estimated 13-fold higher odds of fetal death. An elevated ratio identified a small subgroup of participants with angiogenic imbalance and a substantially higher risk of stillbirth, as reflected by a high PPV, although the wide CIs may have been due to the small number of positive tests. The moderate NPV for both cutoffs may indicate that a low ratio provides limited reassurance to rule out the risk of stillbirth. These findings suggest that angiogenic imbalance, a marker of placental dysfunction, may play an important role in the pathogenesis of stillbirth in low-resource settings.

Prior studies have shown that the sFlt-1:PlGF ratio is predictive of preeclampsia,^9,10,11^ as well as of early-onset FGR and SGA.^12^ However, evidence of an association of angiogenic markers with stillbirth has been limited and primarily derived from clinical populations or late-pregnancy assessments. In a multicenter study of women with suspected preeclampsia, a major contributor to pregnancy-associated morbidity and mortality, an sFlt-1:PlGF ratio cutoff of 38 was identified as a useful marker of the short-term absence of preeclampsia in women with singleton pregnancies.^11^ Between 20 and 34 weeks’ gestation, a ratio less than 33 has been associated with a low risk of preeclampsia, whereas a ratio greater than 85 has been associated with a high risk of preeclampsia.^19^ In a prospective study of 467 women with preeclampsia, higher sFlt-1:PlGF ratios (≥250) were associated with clinical complications, such as stillbirth; eclampsia; and hemolysis, elevated liver enzymes, and low platelets syndrome, among women with preterm preeclampsia.^10^ In a prospective study among 175 singleton pregnancies with fetal weight less than the 10th percentile diagnosed between 20 and 31 weeks’ gestation, the standard cutoffs (38, 85, and 110) estimated the risk for early-onset FGR and SGA.^12^

The biological mechanism remains incompletely understood, but it might be associated with excess circulating sFlt-1 binding to and neutralizing PlGF, suppressing the proangiogenic signaling that is necessary for placental vascular development.^10,20^ Evidence from mouse models supports that elevated or overexpressed sFlt-1 may disrupt placental vascularization, impair trophoblast differentiation, and result in placental dysfunction and fetuses with FGR.^21,22^ In humans, similar antiangiogenic shifts have been linked to impaired uteroplacental perfusion, leading to microvascular rarefaction and reduced oxygen and nutrient transfer to the fetus.^23^ The resulting fetal hypoxia can trigger oxidative stress and inflammation,^24,25^ ultimately compromising fetal growth and survival.^26^ Thus, a marked elevated sFlt-1:PlGF ratio may reflect a severe placental vascular dysregulation, potentially predisposing to fetal death or FGR. Although we describe potential mechanisms to explain the association between the sFlt-1:PlGF ratio and stillbirth, further studies are needed to clarify the underlying pathways, including the role of clinically recognized preeclampsia.

### Limitations

We acknowledge several limitations. First, the sample size was relatively small given the rarity of stillbirth and may have limited precision for subgroup analyses. Second, angiogenic biomarkers were measured at a single gestational time point; serial measurements may allow better characterization of temporal patterns preceding stillbirth. Third, residual confounding cannot be ruled out despite adjustment for multiple covariates. Fourth, when control participants were randomly selected, we restricted the criteria to full-term live births without low birth weight, SGA, and large for gestational age to limit additional heterogeneity in a relatively small sample. Although this approach was intended to improve comparability, it may have introduced selection bias if the sFlt-1:PlGF ratio was also associated with the excluded outcomes; therefore, we cannot exclude the possibility that our ORs were influenced by this selection. Finally, our findings may not be fully generalizable to other populations, especially high-resource settings, although the biological mechanisms may be conserved across populations. Importantly, because the burden of stillbirth is high in LMICs, our results remain particularly relevant for underrepresented populations who have limited access to advanced diagnostic tools and are disproportionately at risk for placental dysfunction and pregnancy complications.

Although the sFlt-1:PlGF ratio is currently used primarily in the evaluation of suspected preeclampsia, our findings suggest that it may have broader translational relevance. Future studies need to evaluate whether incorporating angiogenic biomarker screening into antenatal care, either alone or within multivariable risk estimation models, may aid in identifying pregnancies at elevated risk for stillbirth and support targeted monitoring, particularly in resource-limited settings.

## Conclusions

This case-control study of an LMIC cohort found that a high sFlt-1:PlGF ratio was associated with stillbirth, underscoring the potential role of angiogenic dysregulation in the pathway leading to fetal death. These findings highlight the need for further translational research to evaluate angiogenic biomarkers as tools for early risk identification. Larger, prospective studies are needed to confirm these results, evaluate potential clinical utility, and inform efforts to reduce the persistent burden of this silent epidemic.

## Notes

Supplement.
Data Sharing Statement
