# Supplementary material for: Maternal Circulating sFlt-1:PlGF Ratio and Stillbirth
Source: JAMA Netw Open. 2026 Apr 17;9(4):e267652. doi: 10.1001/jamanetworkopen.2026.7652 (PMC13090853; doi:10.1001/jamanetworkopen.2026.7652)
Supplement: Supplement. — Data Sharing Statement [file jamanetwopen-e267652-s001.pdf]

Data Sharing Statement

Data
Data available: No

Additional Information
Explanation for why data not available: We will not share the individual-level data from this study because they include sensitive, potentially identifiable clinical information from a relatively small cohort.
